# Facial Mobility after Maxilla-Mandibular Advancement in Patients with Severe Obstructive Sleep Apnea Syndrome: A Three-Dimensional Study

**DOI:** 10.1155/2017/1574304

**Published:** 2017-06-04

**Authors:** Laura Verzé, Francesca Antonella Bianchi, Niccolò Barla, Serena Maria Curti, Giovanni Gerbino, Guglielmo Amedeo Ramieri

**Affiliations:** ^1^Legal Medicine Section, Department of Public Health and Paediatrics, University of Turin, Corso Galileo Galilei 22, 10126 Turin, Italy; ^2^Maxillo-Facial Surgery Section, Department of Surgical Sciences, University of Turin, Corso Dogliotti 38, 10126 Turin, Italy

## Abstract

*Introduction. *The functional results of surgery in terms of facial mobility are key elements in the treatment of patients. Little is actually known about changes in facial mobility following surgical treatment with maxillomandibular advancement (MMA).* Objectives.* The three-dimensional (3D) methods study of basic facial movements in typical OSAS patients treated with MMA was the topic of the present research.* Materials and Methods. *Ten patients affected by severe obstructive sleep apnea syndrome (OSAS) were engaged for the study. Their facial surface data was acquired using a 3D laser scanner one week before (T1) and 12 months after (T2) orthognathic surgery. The facial movements were frowning, grimace, smiling, and lip purse. They were described in terms of surface and landmark displacements (mm). The mean landmark displacement was calculated for right and left sides of the face, at T1 and at T2.* Results. *One year after surgery, facial movements were similar to presurgical registrations. No modifications of symmetry were present.* Conclusions. *Despite the skeletal maxilla-mandible expansion, orthognathic surgical treatment (MMA) of OSAS patients does not seem to modify facial mobility. Only an enhancement of amplitude in smiling and knitting brows was observed. These results could have reliable medical and surgical applications.

## 1. Introduction

Morphological changes after orthognathic surgery involved soft tissue changes in terms of shape and size [1–9].

Many studies have been conducted regarding the modifications of bone structures after maxillofacial surgery in different pathological conditions, but only a few have been conducted about soft tissue changes and, in particular, about facial movements [10].

Because of the importance of facial mimicry in human relationships and the anatomical, physiological, and psychological effects of its alterations, recent studies evaluated the facial mobility of patients undergoing surgical treatment involving facial bones. An accurate analysis of pre- and postsurgical facial movements could be part of a more precise prevision of the surgical treatment and its results [11].

In the present study, the authors aimed to investigate, by the means of three-dimensional (3D) imaging methods, pre- and postsurgical facial movements in patients with severe obstructive sleep apnea syndrome (OSAS) treated with maxilla-mandible advancement (MMA), which is the most successful surgical treatment for these patients, in which the jaw is advanced and, if possible, a stable occlusion maintained [12]. The bimaxillary advancement due to MMA leads to a skeletal expansion, which increases the soft tissue support [13, 14]: this augmentation of facial projection has a positive or neutral effect on facial aesthetics; despite the alteration of cephalometric measurements [13, 15], MMA leads to a sagittal projection of the cheeks, lips, and chin [12]. Most patients who underwent MMA for OSAS noted moderate changes in their facial appearance [15] and the evaluation by 3D laser scanning showed that surgery in OSAS patients did not cause an impairment of facial appearance [12].

The aim of this research was to examine if a group of basic facial movements were modified by bone displacements and scars due to MMA and to evaluate if mimic muscle movements could be modified, or not, by bone displacements and scars from surgery. No studies have ever been conducted on the possible facial muscular movement modifications after MMA.

## 2. Patients and Methods

Ten Caucasian adult patients (mean age 44.9 years, range 33–60 years; eight males, two females), with severe OSAS (Apnea Hypopnea Index, AHI, mean value 69.8 ± 35.2), requiring MMA for its treatment, were included in this prospective study.

Inclusion criteria were middle age, presurgical normal cephalometric measurements, and class I obesity (BMI 30–34.9 kg/m^2^) [17] with mean BMI value: 31.6 kg/m^2^ ± 5.5. The patients who underwent laser scanning both before and after surgery were included in the study.

Exclusion criteria were a previous history of craniofacial injuries, syndromes, or operations. Moreover, patients with dental-skeletal discrepancies leading to facial deformity (mainly severe class II deformities), in which occlusion correction and preoperative orthodontic treatment were incorporated in the treatment plan, were not included in this study (Table 1).

Regarding sex, the different number between male and female subjects was related to the fact that OSAS pathology was prominent in the male sex [18].

All the patients underwent a similar surgical procedure, consisting of a MMA (standardized surgical treatment consisting of a LeFort I osteotomy and bilateral sagittal split-ramous osteotomies), with skeletal advancement planned between 10 and 12 mm [12]. Surgical procedures were performed by the same surgeon, under general anaesthesia, at the Division of Maxillo-Facial Surgery, Department of Surgical Sciences, University of Turin, Turin, Italy. The surgeon was a coauthor.

Informed consent was obtained from all participants. This study was performed in agreement with local institutional review board. The Helsinki Declaration guidelines were followed.

### 2.1. Data Registration

Facial surface data was acquired one week before (T1) and 12 months after surgery (T2), using a Cyberware 3030 RGB (Cyberware, Inc., 2110, Del MonteAvenue, Monterey, California, 93940 USA) laser scanner by means of a specific protocol [6, 7] with the head oriented in the natural head position (NHP).

All the faces were firstly registered in the rest position at NHP and then the patients were asked to assume five basic facial expressions: frowning, knitting brows, grimace, smiling, and lip purse at T1 and T2.

The attained data were transferred to a graphics workstation for elaboration with Cyberware Echo software (Cyberware Inc.).

3D facial surfaces were reconstructed and scan shell alignment (face model) and comparison were carried out using Rapid Form 2004 software (INUS Technologies Inc., 2004, Seoul, South Korea).

The accuracy of this method and its reliability in studying facial movements have been previously described [19, 20].

### 2.2. Facial Surfaces Superimposing

Morphologic changes of facial mobility were investigated as facial surfaces superimposing (“shell-to-shell” deviation). For each patient, facial scans at T1 and T2 were registered on homologous points. The 3D registration of every facial movement was recorded on its corresponding shell in the rest position using an alignment of manually selected corresponding landmarks and/or anatomical areas that were identified among those which were not modified by surgery and remained immobile during each studied movement. The software automatically recorded the two 3D images to obtain the best fit between their surfaces using the given points.

**Figure 1:**
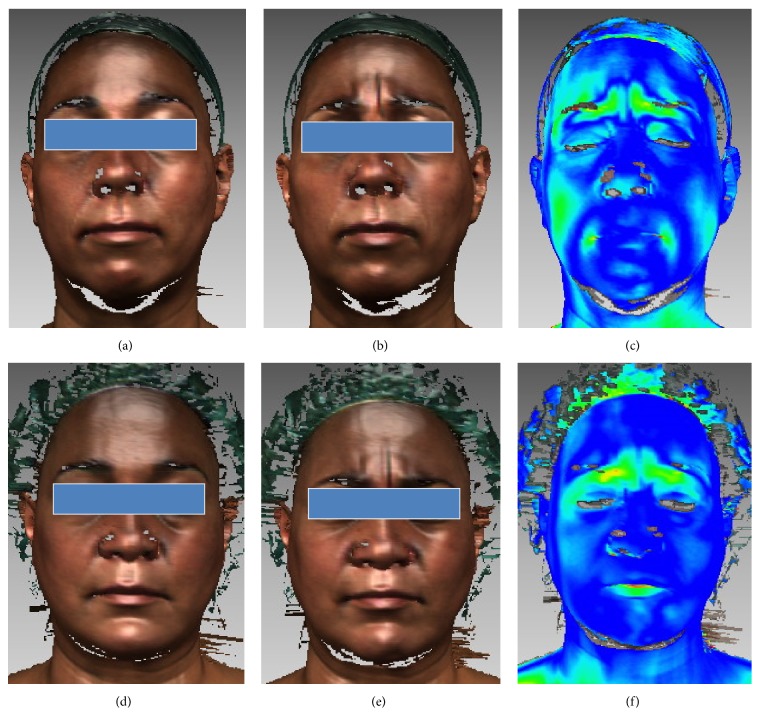
Graphics program: Rapidform 2004, MSOffice 2013. 3D scanning registrations at T1 (a, b, c); 3D scanning registrations at T2 (d, e, f). Clearance vector map ((c) and (f)) obtained by the superimposition, respectively, of (a) with (b) and (d) with (e). Note the reduction of the associated movements in T2 (f).

**Figure 2:**
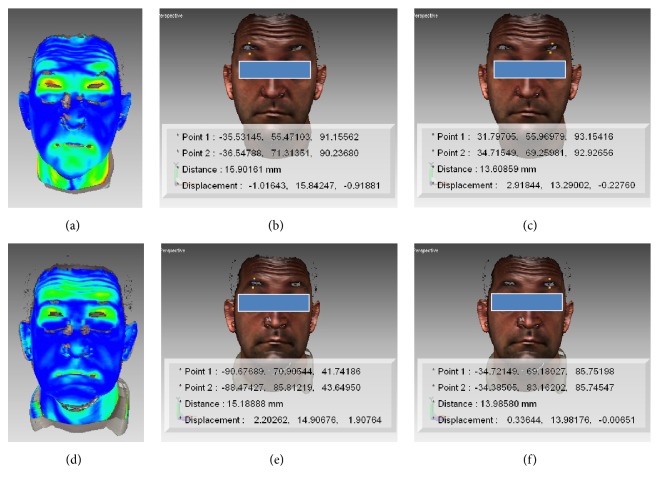
Graphics program: Rapidform 2004, MSOffice 2013. Frowning: clearance vector mapping at T0 (a) and T1 (d). Point to point distance (mm) of superciliare (sci) at right ((b) and (e)) and at left ((c) and (f)) side. Note the reduction of the associated movements in the region of the inferior 2/3 of the face during the movement at T1.

**Figure 3:**
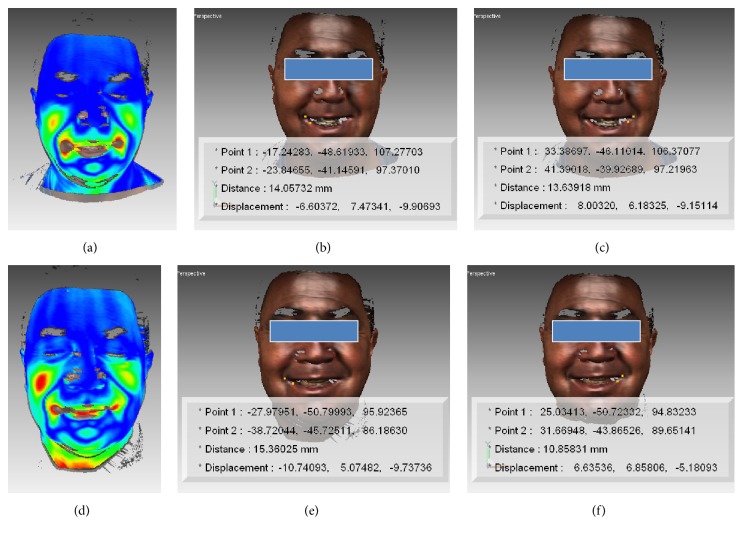
Graphics program: Rapidform 2004, MSOffice 2013. Smiling: clearance vector mapping at T0 (a) and T1 (d). Point to point distance (mm) of cheilion (ch) at right ((b) and (e)) and at left ((c) and (f)) side. Note the main amplitude of the movement at T1.

The program generated a colour scale indicating the areas with different degree of tissue displacement. This process provided information on regional changes following every movement at T1 and T2 that were visualised as a pseudo-colour map (shell-to-shell deviation, clearance vector map) (Figures 1, 2(a), 2(d), 3(a), and 3(d)).

A colour scale (clearance vector map: CVM) indicated the areas with different degrees of displacement of the two superimposed surfaces: in red, the areas with major movement, in green minor movement, and in blue no movement (Figures 1(c), 1(f), 2(a), 2(d), 3(a), and 3(d)).

### 2.3. Landmark Displacement

Landmark displacements were measured independently by two operators. The reliability of the method and mean error has been previously studied [18].

For data elaboration, anatomical points chosen from classical anthropometry [20] were taken in consideration: superciliare (sci) for frowning and knitting brows, alar curvature point (ac) for grimace, and cheilion (ch) for smile and lip purse (Table 2). These points were considered “tracing landmarks” for the movements required [11, 19, 21].

For every facial movement, we measured the displacement in millimeters of every tracing landmark at T1 and T2 on right and left sides of the face. Mean and standard deviations were calculated and, subsequently, statistical analysis was carried out with the paired *t*-test of the two groups, and it was used to assess differences between T1 and T2, for every facial movement. The level of significance was set at *p* < 0.05 (Table 3).

## 3. Results

### 3.1. Facial Surfaces Superimposing

Surgery did not seem to substantially modify the appearance of the facial mobility of every subject. The shell-shell deviation map did not indicate asymmetries between the left and right sides of the face after surgery (Figures 2(a), 2(d), 3(a), and 3(d)).

Clearance vector mapping revealed the presence of “associated” atypical movements, unrelated to specific muscle activation involved in the execution of the requested facial movements [16, 19, 21]. In two cases their presence was reduced at T2 (Figures 1(c), 1(f), 2(a), and 2(d)).

In T2 in comparison to T1, an enhancement of knitting brows and smiling amplitude was present (Figure 3) (Table 3).

### 3.2. Landmark Displacement

Correspondence was observed between the landmark displacements measure and the clearance vector map. The landmark displacement study confirmed shell-to-shell deviation gross observation (Table 3).

No differences between the left and the right side were noted, one year after surgery.

During knitting brows and smiling a significant enhancement of the movement was observed at T2 in comparison to T1.

No significant differences in facial morphological registration were observed at the two different times of the registrations. These results were the same as previous studies conducted on another group of bimaxillary class III patient's surgery [11].

## 4. Discussion

In this paper, the authors presented a 3D study of facial mobility before and after maxilla-mandibular advancement (MMA) in patients with severe obstructive sleep apnea (OSAS) [12].

3D laser scanning analysis of the face was a valuable method for studying facial morphology and mobility and it might provide valuable information on the effects on soft tissues of orthodontic and surgical treatments [1, 4, 12, 18, 22–26].

Previous studies were conducted on facial mobility with 3D methods [20, 21, 27, 28]. A few previous studies were conducted about facial mobility after bone surgery [11] and little is actually known about changes in facial mobility following MMA surgery in OSAS patients.

The first result of the present study was to note that this invasive surgical treatment did not significantly modify facial movements: 3D analysis of facial movements showed that one year after surgery facial mobility was very similar to before surgery.

In our previous study conducted on normal people and in child facial mobility clearance vector map analysis showed that each movement led to similar soft tissue deformation in the same facial areas in all subjects. Moreover, clearance vector mapping revealed the presence of “associated,” atypical movements during the execution of the requested facial movements [20].

In the current study, except for a few cases, surgery did not modify this type of movement. The authors also observed that accessory movements were present in all the OSAS patients at presurgical control, while they appeared to be reduced in a few cases one year after surgery (Figures 1(c), 1(f), 2(a), and 2(d)). On the basis of this observation we could suggest that MMA, modifying respiratory movements (breath activity) and consequently facial muscles contracture, could in some cases meliorate and reduce also facial muscle activity. Moreover, in the absence of neurological disease, a type of memory for face mobility concerning the neural processing of moving faces might also be considered [29].

After surgery, an enhancement of knitting brows and smiling was observed. The increasing of smiling was previously described in subjects surgically treated with bimaxillary surgery in class III patients [11]. It is known that the most common facial expressions involve the areas of the eyebrows and the mouth [30].

In the same way in previous studies conducted with 3D methods after bimaxillary surgery in class III patients, adults undergoing reconstructive facial surgery retained the same mimic movements one year after surgery, despite the changes to the underlying bones, except for smiling and knitting brows [11]. This result was similar to the present one, also if the surgical treatment was not the same. Moreover, other studies indicated that facial mobility was not very much influenced; that is, also sex and age had a limited influence on total facial motion and asymmetry in normal adult men and women [31].

Previous 3D studies showed that orthognathic operations improved facial symmetry. Patients in the maxillary advancement alone group did not show a significant improvement in facial symmetry after operation. The general facial asymmetry scores showed no significant change after operation in the class II bimaxillary group [1]. Facial mobility in the current study seems not to be dependent on the bone displacement. Only in smiling we show a significant difference between right and left side. However, this data should be consequent to the greater mobility of the face in this side, and not dependent on bone displacement after surgery.

Limitations of the current study were the small sample size and the discrepancy of number between males and females. OSAS pathology was prominent in the male sex [18] and we had only two female subjects for the study. Further studies are necessary for the knowledge of facial movements after MMA surgery.

The knowledge of the possible modifications of facial expression and mimicry conservation in pre- and postsurgical patients is an important goal in maxilla-facial surgery for the programming of the postsurgical results, in particular if bone displacements are involved.

3D laser scanning analysis of the face was a valuable method for studying facial morphology and mobility and might provide valuable information on the effects on soft tissues of orthodontic and surgical treatments [4, 5, 22–26].

The topic of this study may be of current interest for the medical community and especially for oral and maxilla-facial surgeons and medical examiners for facial identification.

## 5. Conclusions

3D analysis of soft tissues using laser scanning is a valuable method for the study of facial mobility. From the current results MMA did not substantially modify facial mobility in terms of facial expression, and one year after surgery, mimicry was very similar to before surgery. After MMA, an enhancement of knitting brows and smiling was present. No asymmetries were observed.

## Figures and Tables

**Table 1:** Comparison between pre- and post-op BMI, polysomnographics, and cephalometrics measurements.

	Normal values	Pre-op	Post-op
BMI (kg/m^2^)	18.5–24.9	31.6 ± 5.5	28 ± 1.4
PSG			
RDI (hr)		74.1 ± 34.4	10.3 ± 7.2
ODI (%)		59.5 ± 25.3	9.1 ± 8
AHI (events/hr)	<5	69.8 ± 35.2	17.3 ± 16.7
L-cephalometry			
SNA (°)	82 ± 3.5	82.5 ± 2.9	87.7 ± 3.7
SNB (°)	80.9 ± 3.4	78.7 ± 3.2	82.1 ± 3
MP-H (mm)	15.4 ± 3	27 ± 3.6	23.2 ± 2.7
PAS (mm)	12.8 ± 3.2	6.7 ± 2	14.1 ± 1.9

PSG, polysomnography recordings; BMI, body mass index; RDI, respiratory disturbance index; ODI, oxygen desaturation index; AHI, apnea-hypopnea index; SNA, angle from Sella-Nasion-point A; SNB, angle from Sella-Nasion-point B; MP-H, distance from mandibular plane to hyoid bone; PAS, posterior airway space.

**Table 2:** Landmarks employed for the study and their definitions [16].

Landmarks	Abbreviations	Definitions
Superciliare	Sci	The highest point on the upper borderline in the mid-portion of each eyebrow.
Alar curvature point	Ac	The facial insertion of the alar base.
Cheilion	Ch	The point located at each labial commissure.

**Table 3:** Landmarks displacement (in millimeters) in the right and left side of the face at T1 and T2.

Movements	Landmarks	Side	Mean and Std Dev	Mean and Std Dev	Δ*µ*	*p* value
			T1	T2		
Frowning	sci	Right	10.90 ± 4.17	8.82 ± 4.89	2.08	*p* < 0.50
	sci	Left	11.25 ± 3.82	9.06 ± 4.39	2.19	*p* < 0.50
Knitting brows	sci	Right	3.98 ± 1.21	5.57 ± 1.68	−1.59	**p** < 0.015^**∗**^
	sci	Left	3.95 ± 1.04	5.51 ± 1.66	−1.56	**p** < 0.015^**∗**^
Grimace	ac	Right	6.99 ± 3.30	6.59 ± 3.29	0.39	*p* < 0.50
	ac	Left	7.30 ± 3.29	6.39 ± 3.39	0.91	*p* < 0.50
Smiling	ch	Right	11.89 ± 3.93	16.36 ± 6.00	−4.47	**p** < 0.003^**∗**^
	ch	Left	11.03 ± 4.21	15.32 ± 6.55	−4.30	**p** < 0.003^**∗**^
Lip purse	ch	Right	12.88 ± 1.97	12.77 ± 2.09	0.11	*p* < 0.40
	ch	Left	12.18 ± 1.62	13.52 ± 3.33	−1.34	*p* < 0.15

Mean and Std Dev (standard deviation) are calculated at T1 and T2. Medium distance (Δ*µ*) between homologous points is considered between T1 and T2 in absolute term. *p* value is calculated according to Student's *t*-distribution and it considers the *t* critical value in one-tail case. Statistically significant differences were considered for *p* < 0.05 (^**∗**^bold). The landmarks used were superciliare (sci) for frowning and knitting brows, alar curvature point (ac) for grimace, and cheilion (ch) for smile and lip purse.

## References

[1] Hajeer M. Y., Ayoub A. F., Millett D. T. (2004). Three-dimensional assessment of facial soft-tissue asymmetry before and after orthognathic surgery. *British Journal of Oral and Maxillofacial Surgery*.

[2] Ramieri G. A., Nasi A., Dell'Acqua A., Verzé L. (2008). Facial soft tissue changes after transverse palatal distraction in adult patients. *International Journal of Oral and Maxillofacial Surgery*.

[3] Altug-Atac A. T., Atac M. S., Kurt G., Karasud H. A. (2010). Changes in nasal structures following orthopaedic and surgically assisted rapid maxillary expansion. *International Journal of Oral and Maxillofacial Surgery*.

[4] O'Grady K. F., Antonyshyn O. M. (1999). Facial asymmetry: Three-dimensional analysis using laser surface scanning. *Plastic and Reconstructive Surgery*.

[5] McCance A. M., Moss J. P., Fright W. R., James D. R., Linney A. D. (1992). A three dimensional analysis of soft and hard tissue changes following bimaxillary orthognathic surgery in skeletal III patients. *British Journal of Oral and Maxillofacial Surgery*.

[6] McCance A. M., Moss J. P., Wright W. R., Linney A. D., James D. R. (1992). A three-dimensional soft tissue analysis of 16 skeletal class III patients following bimaxillary surgery. *British Journal of Oral and Maxillofacial Surgery*.

[7] Moss J. P., McCance A. M., Fright W. R., Linney A. D., James D. R. (1994). A three-dimensional soft tissue analysis of fifteen patients with class II, division 1 malocclusions after bimaxillary surgery. *American Journal of Orthodontics and Dentofacial Orthopedics*.

[8] Baik H.-S., Kim S.-Y. (2010). Facial soft-tissue changes in skeletal Class III orthognathic surgery patients analyzed with 3-dimensional laser scanning. *American Journal of Orthodontics and Dentofacial Orthopedics*.

[9] Shimomatsu K., Nozoe E., Ishihata K., Okawachi T., Nakamura N. (2012). Three-dimensional analyses of facial soft tissue configuration of Japanese females with jaw deformity—a trial of polygonal view of facial soft tissue deformity in orthognathic patients. *Journal of Cranio-Maxillo-Facial Surgery*.

[10] Ko E. W.-C., Huang C. S., Lo L., Chen Y. (2012). Longitudinalobservation of mandibularmotion pattern in patients with skeletal class III malocclusionsubsequent to orthognathic surgery. *Journal of Oral and Maxillofacial Surgery*.

[11] Verzé L., Bianchi F. A., Dell'Acqua A., Prini V., Ramieri G. A. (2011). Facial mobility after bimaxillary surgery in class III patients: a three-dimensional study. *Journal of Craniofacial Surgery*.

[12] Gerbino G., Bianchi F. A., Verzé L., Ramieri G. (2014). Soft tissue changes after maxillo-mandibular advancement in OSAS patients: A three-dimensional study. *Journal of Cranio-Maxillofacial Surgery*.

[13] Li K. K., Riley R. W., Powell N. B., Guilleminault C. (2000). Maxillomandibular advancement for persistent obstructive sleep apnea after phase I surgery in patients without maxillomandibular deficiency. *Laryngoscope*.

[14] Blumen M. B., Buchet I., Meulien P., Hauw C. H., Neveu H., Chabolle F. (2009). Complications/adverse effects of maxillomandibular advancement for the treatment of OSA in regard to outcome. *Otolaryngology - Head and Neck Surgery*.

[15] Li K. K., Riley R. W., Powell N. B., Guilleminault C. (2001). Patient's perception of the facial appearance after maxillomandibular advancement for obstructive sleep apnea syndrome. *Journal of Oral and Maxillofacial Surgery*.

[16] Farkas L. G. (1994). *Anthropometry of the Head and Face*.

[17] National Institutes of Health (1998). Clinical guidelines on the identification, evaluation, and treatment of overweight and obesity in adults: the evidence report. *Obesity Research*.

[18] Tsai H.-H., Ho C.-Y., Lee P.-L., Tan C.-T. (2009). Sex differences in anthropometric and cephalometric characteristics in the severity of obstructive sleep apnea syndrome. *American Journal of Orthodontics and Dentofacial Orthopedics*.

[19] Ramieri G. A., Spada M. C., Nasi A. (2006). Reconstruction of facial morphology from laser scanned data. Part I: Reliability of the technique. *Dentomaxillofacial Radiology*.

[20] Verzé L., Nasi A., Quaranta F., Vasino V., Prini V., Ramieri G. (2011). Quantification of facial movements by surface laser scanning. *Journal of Craniofacial Surgery*.

[21] Sidequersky F. V., Verzé L., Mapelli A., Ramieri G. A., Sforza C. (2014). Quantification of facial movements by optical instruments: Surface laser scanning and optoelectronic three-dimensional motion analyzer. *Journal of Craniofacial Surgery*.

[22] Bush K., Antonyshyn O. (1996). Three-dimensional facial anthropometry using a laser surface scanner: Validation of the technique. *Plastic and Reconstructive Surgery*.

[23] Ismail S. F. H., Moss J. P. (2002). The three-dimensional effects of orthodontic treatment on the facial soft tissues - A preliminary study. *British Dental Journal*.

[24] Kusnoto B., Evans C. A. (2002). Reliability of a 3D surface laser scanner for orthodontic applications. *American Journal of Orthodontics and Dentofacial Orthopedics*.

[25] Nooreyazdan M., Trotman C.-A., Faraway J. J. (2004). Modeling facial movement: II. A dynamic analysis of differences caused by orthognathic surgery. *Journal of Oral and Maxillofacial Surgery*.

[26] Bianchi F. A., Verzé L., Ramieri G. (2012). Three-dimensional surface laser scanner for planning and outcome evaluation in orthognatic surgery. *Italian Journal of Maxillofacial Surgery*.

[27] Mehta R. P., Zhang S., Hadlock T. A. (2008). Novel 3-D video for quantification of facial movement. *Otolaryngology - Head and Neck Surgery*.

[28] Lübbers H.-T., Medinger L., Kruse A. L., Grätz K. W., Obwegeser J. A., Matthews F. (2012). The influence of involuntary facial movements on craniofacial anthropometry: A survey using a three-dimensional photographic system. *British Journal of Oral and Maxillofacial Surgery*.

[29] Roark D. A., Barrett S. E., Spence M. J., Abdi H., O'Toole A. J. (2003). Psychological and neural perspectives on the role of motion in face recognition. *Behavioral and cognitive neuroscience reviews*.

[30] Taylor K. T. (2001). *Forensic Art and Illustration*.

[31] Sforza C., Mapelli A., Galante D. (2010). The effect of age and sex on facial mimicry: a three-dimensional study in healthy adults. *International Journal of Oral and Maxillofacial Surgery*.

